# Dressler's syndrome demonstrated by late gadolinium enhancement cardiovascular magnetic resonance

**DOI:** 10.1186/1532-429X-11-23

**Published:** 2009-07-23

**Authors:** Christopher D Steadman, Jeffrey Khoo, Jan Kovac, Gerry P McCann

**Affiliations:** 1 University Hospitals of Leicester, Glenfield Hospital, Groby Road, Leicester, LE3 9QP, UK

## Abstract

A 49-year old patient presented late with an anterolateral ST-elevation myocardial infarction and was treated with rescue angioplasty to an occluded left anterior descending artery. Her recovery was complicated by low-grade pyrexia and raised inflammatory markers. Cardiovascular magnetic resonance 5 weeks after the acute presentation showed transmural infarction and global late gadolinium enhancement of the pericardium in keeping with Dressler's syndrome.

## Background

Secondary pericarditis can occur following myocardial infarction; this is relatively common acutely between 2 and 4 days after myocardial infarction. In contrast Dressler's syndrome presents with a low-grade fever and chest pain 2 to 10 weeks after myocardial infarction, and is less common, affecting between 1 and 5% of patients [1-4]. In Dressler's initial description the cause was hypothesised to be irritation due to the presence of blood in the pericardial space[5], however several possible pathogenic mechanisms have been proposed since including; autosensitisation to myocardial antigens released into the circulation during infarction[6], latent viruses[7] or simply that the syndrome represents a prolonged and exaggerated form of early post-infarction pericarditis[8]. Since the introduction of reperfusion, with thrombolysis and balloon angioplasty, the incidence of DS has decreased[9,10]. It is postulated that the diminution of the infarct size and the shortened time of exposure of myocardial antigens to the immune system may be responsible[10]. However it has been suggested that the immunomodulatory properties of modern post-myocardial infarction drug therapies, such as ACE inhibitors, statins and B-blockers, may play a role[11]. The clinical presentation can be classical, with pleuritic pain and an associated pericardial rub; however the differential diagnosis includes further acute coronary syndrome and pulmonary embolism which may result in the need for further diagnostic investigation. We describe a case where global pericardial inflammation late post-myocardial infarction is clearly demonstrated by late gadolinium enhancement (LGE) cardiovascular magnetic resonance (CMR).

## Case presentation

A 49 year old woman presented with jaw and arm pain to her local Emergency Department. She had the pain since the previous evening, approximately 14 hours, and had initially presented to her dentist before being redirected. Cardiac risk factors included hypertension, smoking and a positive family history of ischaemic heart disease in her father. The ECG showed anterolateral ST elevation and because of this and ongoing pain, she was thrombolysed with retaplase despite the late presentation. At 90 minutes the ECG changes and pain had not resolved and therefore she was transferred to our tertiary centre for rescue angioplasty. The left anterior descending artery (LAD) was occluded with a large thrombus load. The LAD was successfully opened and a Vision 3.5 × 15 mm bare metal stent was deployed, the procedure was covered with heparin and abciximab and an intra-aortic balloon pump. There was moderate circumflex and right coronary artery disease which was not intervened upon. Cardiac enzymes were elevated with peak CK of >4000 iu/L and troponin I of 98 μg/L. A transthoracic echocardiogram the following day showed a mildly dilated left ventricle (LV) with mild hypertrophy. The anterolateral wall was akinetic with moderate to severely impaired LV function. There was no significant valvular disease and a 1 cm pericardial effusion around the posterior, inferior and lateral walls and right heart with no haemodynamic compromise. Her recovery was complicated by low-grade pyrexia and raised inflammatory markers, peak CRP 321 mg/L. Blood, urine and sputum cultures were negative however she was treated empirically for a chest infection with doxycycline. An episode of hypoxia and chest pain without gross changes on chest radiograph resulted in a CT pulmonary angiogram which did not show any evidence of pulmonary embolism but some basal atelectasis. Two weeks after admission she was asymptomatic. Prior to discharge an exercise tolerance test was arranged to assess for ischaemia in view of the moderate untreated coronary disease. She completed 6 minutes of the Bruce protocol achieving 85% of her maximal predicted heart rate, there was no pain but mild ST depression inferiorly. The test was submaximal but did not show gross ischaemia. In view of this she was discharged home with a plan for outpatient myocardial perfusion imaging.

**Figure 1:**
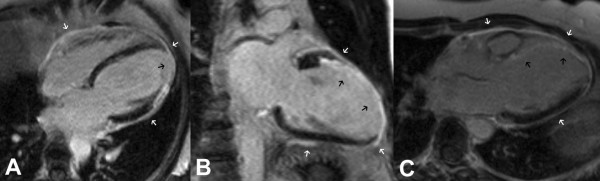
**LGE of the infarcted anterior, anteroseptal and anterolateral wall (black arrows)**. (A) 4-chamber, (B) 2-chamber and (C) 3-chamber views. LGE of the pericardium can also be seen (white arrows).

**Figure 2:**
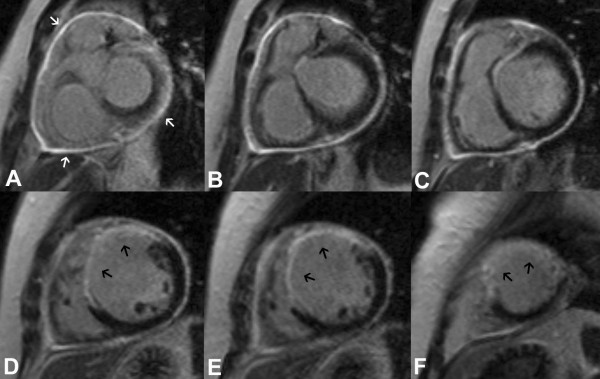
**Global LGE of the pericardium on short axis views from base to apex (A-F), marked with white arrows on first slice**. LGE of the infarcted anterior and anteroseptal walls extending into lateral wall towards apex (black arrows).

**Figure 3:**
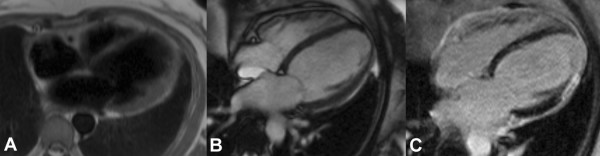
**4-chamber view demonstrating global pericardial inflammation with no obvious pericardial thickening; (A) HASTE images, (B) FISP images, (C) LGE**.

**Figure 4:**
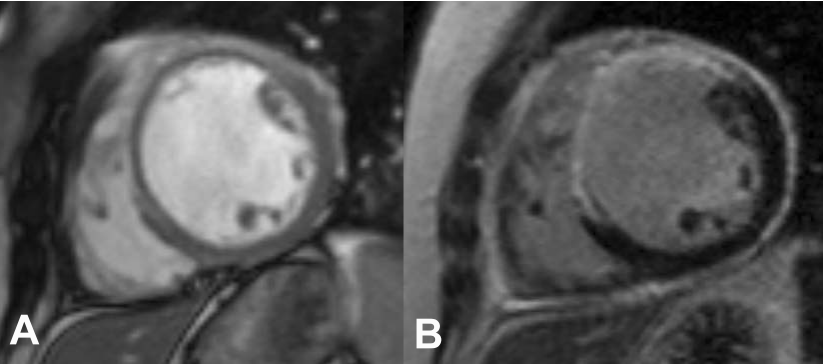
**Short axis view demonstrating global pericardial inflammation with no obvious pericardial thickening; (A) FISP images, (B) LGE**.

She attended for a CMR stress perfusion study 5 weeks after her initial infarct. At this point she did not report any symptoms. The LV was moderately dilated with an akinetic anteroseptal wall (Additional files 1 and 2). LGE images demonstrated extensive transmural anteroseptal and apical myocardial infarction (Figure 1). There was moderate functional mitral regurgitation. Stress perfusion did not demonstrate any reversible ischaemia. LGE images also showed striking global pericardial inflammation (Figure 2). There was no obvious pericardial thickening demonstrable on long axis (Figure 3) or short axis imaging (Figure 4).

## Discussion

This patent presented late after ST elevation myocardial infarction with a resulting large rise in cardiac enzymes and significant transmural myocardial infarction. Her inpatient stay was complicated by low-grade pyrexia, raised inflammatory markers and a small pericardial effusion. In view of the negative cultures and subsequent CMR findings this was likely to have been due to pericardial inflammation. CMR is one of the most versatile modalities to assess pericardial disease[12]. Pericardial inflammation has been demonstrated by LGE with excellent sensitivity and specificity [13]. CMR signs of pericarditis have been reported in up to 40% of patients post-myocardial infarction when imaged early, 6.1 ± 2.2 days, and this tends to be localised at the site of infarction [14]. The CMR in this case was performed 5 weeks after the acute presentation. This case is unique in that it demonstrates global pericardial inflammation late post-myocardial infarction and given the clinical context is consistent with a diagnosis of Dressler's syndrome. Whether such findings are commonplace is unclear given CMR is not usually requested for this indication. This case demonstrates that global pericardial inflammation in Dressler's syndrome can be clearly visualised by LGE on CMR. This may prove clinically useful when trying to establish a cause for atypical post-myocardial infarction pain without requiring exposure to invasive procedures or ionizing radiation.

## Consent

Written informed consent was obtained from the patient for publication of this case report and accompanying images. A copy of the written consent is available for review by the Editor-in-Chief of this journal.

## Competing interests

The authors declare that they have no competing interests.

## Authors' contributions

CS drafted the manuscript and manipulated the CMR images for inclusion. JKhoo helped draft the manuscript. JKovac is the cardiologist responsible for the patient's care. GM acquired and interpreted the CMR images and helped draft the manuscript. All authors read and approved the final manuscript.

## Supplementary Material

Additional file 1 **4-chamber view**. SSFP cine demonstrating moderate LV dilatation, apical dyskinesis and functional mitral regurgitation. Click here for file

Additional file 2 **Short axis view**. SSFP cine at mid-ventricular level demonstrating thinned and akinetic anterior and anteroseptal segments. Click here for file

## References

[B1] Davidson C, Oliver MF, Robertson RF (1961). Post-myocardial-infarction syndrome. Br Med J.

[B2] Dressler W (1959). The post-myocardial-infarction syndrome: a report on forty-four cases. AMA Arch Intern Med.

[B3] Northcote RJ, Hutchison SJ, McGuinness JB (1984). Evidence for the continued existence of the postmyocardial infarction (Dressler's) syndrome. Am J Cardiol.

[B4] Welin L, Vedin A, Wilhelmsson C (1983). Characteristics, prevalence, and prognosis of postmyocardial infarction syndrome. Br Heart J.

[B5] Dressler W (1956). A post-myocardial infarction syndrome; preliminary report of a complication resembling idiopathic, recurrent, benign pericarditis. J Am Med Assoc.

[B6] Earis JE, Marcuson EC, Bernstein A (1985). Complement activation after myocardial infarction. Chest.

[B7] Kossowsky WA, Lyon AF, Spain DM (1981). Reappraisal of the postmyocardial infarction Dressler's syndrome. Am Heart J.

[B8] Gregoratos G (1990). Pericardial involvement in acute myocardial infarction. Cardiol Clin.

[B9] Lichstein E, Arsura E, Hollander G, Greengart A, Sanders M (1982). Current incidence of postmyocardial infarction (Dressler's) syndrome. Am J Cardiol.

[B10] Shahar A, Hod H, Barabash GM, Kaplinsky E, Motro M (1994). Disappearance of a syndrome: Dressler's syndrome in the era of thrombolysis. Cardiology.

[B11] Bendjelid K, Pugin J (2004). Is Dressler syndrome dead?. Chest.

[B12] Bogaert J, Francone M (2009). Cardiovascular magnetic resonance in pericardial diseases. J Cardiovasc Magn Reson.

[B13] Taylor AM, Dymarkowski S, Verbeken EK, Bogaert J (2006). Detection of pericardial inflammation with late-enhancement cardiac magnetic resonance imaging: initial results. Eur Radiol.

[B14] Hombach V, Grebe O, Merkle N, Waldenmaier S, Hoher M, Kochs M (2005). Sequelae of acute myocardial infarction regarding cardiac structure and function and their prognostic significance as assessed by magnetic resonance imaging. Eur Heart J.

